# Virtual versus Physical Channel for Sex Networking in Men Having Sex with Men of Sauna Customers in the City of Hong Kong

**DOI:** 10.1371/journal.pone.0031072

**Published:** 2012-02-10

**Authors:** Shui-Shan Lee, Agnes N. S. Lam, Chi-Kei Lee, Ngai-Sze Wong

**Affiliations:** Stanley Ho Centre for Emerging Infectious Diseases, The Chinese University of Hong Kong, Prince of Wales Hospital, Shatin, Hong Kong; Public Health Agency of Barcelona, Spain

## Abstract

**Background:**

Advances in communication technology may affect networking pattern, thereby influencing the dynamics of sex partnership. The aim of the study is to explore the impacts of partner sourcing through internet and related channels on exposure risk to sexually transmitted infections (STI) including HIV.

**Methods:**

Using venue-based sampling, a cross-sectional questionnaire survey was conducted at saunas frequented by men having sex with men (MSM) in Hong Kong. Comparison was made between MSM sourcing partners through physical venues alone versus concomitant users of physical and virtual channels, the latter referring to internet and smart-phone applications, using bivariate logistic regression.

**Results:**

Over a 7-week study period, 299 MSM were recruited from 9 saunas. Three main types of sex partners were distinguished: steady (46.8%), regular (26.4%) and casual (96.0%) partners. Users of sauna (n = 78) were compared with concomitant users of saunas and virtual channels (n = 179) for partner sourcing. Sauna-visiting virtual channel users were younger and inclined to use selected physical venues for sourcing partners. Smart-phone users (n = 90) were not different from other internet-users in terms of age, education level and single/mixed self-identified body appearance. Classifying respondents into high risk and low risk MSM by their frequency of condom use, concomitant use of both sauna and virtual channels accounted for a higher proportion in the high risk category (71.6% vs. 58.2%, OR = 1.81, p<0.05). In virtual channel users, partner sourcing through smart-phone was not associated with a higher practice of unprotected sex.

**Conclusion:**

MSM sauna customers commonly use virtual channels for sex partner sourcing. Unprotected sex is more prevalent in sauna customers who use virtual channel for sex partner sourcing. While the popularity of smart-phone is rising, its use is not associated with increased behavioural risk for HIV/STI transmission.

## Introduction

In USA and Western Europe, the first wave of human immunodeficiency virus (HIV) epidemics occurred in men having sex with men (MSM) in the early 1980s [1]–[2]. Virus transmission slowed down in the ensuing decade following concerted efforts in safer sex promotion in MSM communities. Paralleled by access to effective highly active antiretroviral therapy (HAART), the overall outlook of HIV/AIDS, in prevention and control contexts, has significantly improved. In the last decade, however, a second wave of HIV spread in MSM was observed [3]–[4], alongside the re-emergence of syphilis, hepatitis C virus (HCV) infection and other sexually transmitted infections (STI) in MSM [5] [6] [7]. Reports of STI in HIV infected MSM constituted an emerging condition of public health concern [8]–[10]. A similar biphasic phenomenon has also been reported in Asian cities, about 5 to 10 years after that in Western countries [11]–[12]. While suboptimal condom use at individual level is often perceived to have ultimately caused the problem, the sequential recurrence of STI transmission in MSM globally suggests that there could be complex dynamic interactions between various factors in shaping the current epidemiologic pattern.

Knowingly, efficient networking is an important facilitating factor for increased sex partnership in MSM [13]. Over the last decades, the widespread use of internet may have facilitated the exposure of MSM to HIV and/or STI. Studies have suggested that internet users were not only younger, but more likely to have practised unsafe sex, a phenomenon reported in Western countries [13] [14] as well as Asian communities, including Hong Kong [15] [16]. Prior to the second wave of HIV spread in MSM, the prevalence of HIV and STI has been on the declining trend. Increased exposure to HIV, if any, is unlikely to be due solely to an overall rise of HIV prevalence in the MSM communities. A recent study suggested that some risk-taking MSM may function as efficient vehicles for STI transmission, if they occupy a bridging position in the network of the community [17]. The connectivity pattern of MSM could therefore be an important determinant, as inferred in the results of our recent study reporting a rising density of the social networks of those who subsequently became HIV infected [18], during which internet has become increasingly used for partner sourcing [19]. The recent rise of HIV prevalence in MSM around the world may have been a result of the interplay between individual risk behaviour as well as social connectivity of the community.

In the last decade, advances in communication technology have moved so rapidly that the convenience and efficiency of the internet for social networking is gradually being overtaken by 3G smart-phones [20], which are handheld devices which combine the functions of personal computing, broadband internet access and mobile phones. Currently, there are an estimated 3.5 billion mobile phone users globally, the number of which is anticipated to reach 6 billion by 2013 [20] [21]. In a relatively short period of time, smart-phones have penetrated significantly in the society. Mobile phone teledensity (i.e. number of phones per person) has reached above 100% in many developed countries [20]. The efficiency of networking is enhanced as the handheld devices can also allow location of the closest possible contacts with GPS applications. It is crucial that the networking dynamics of MSM is tracked in order that their risk can be assessed in context of the changing environment of interpersonal communication, which varies also from city to city. In this study we aimed to explore the impact of partner sourcing through internet and related channels on exposure risk to STI/HIV in the Chinese city of Hong Kong. This was underpinned by the objectives of investigating the prevalence of the use of virtual channel for sex-networking in a cohort of sauna-visiting MSM, and examining potential factors which may be associated with the practice of unsafe sex as defined by the inconsistent use of condom during sexual intercourse with different types of sex partner.

## Methods

### Ethics statement

This study was approved by the Survey and Behavioural Research Ethics Committee of the Chinese University of Hong Kong. Written informed consent was obtained from all participants.

A questionnaire survey was conducted through face-to-face interviews at saunas frequented by MSM in Hong Kong, after venue-based sampling. To begin, mapping of saunas frequented by MSM was performed and an estimation of the number of potential MSM customers at each location and time made. Sauna operators were approached to secure their support in the administration of the surveys at the venues. Two experienced peer workers familiar with the MSM community were trained to conduct the survey at saunas with the permission of the operators. The interviews were conducted using a questionnaire form that had been field tested, with responses recorded on paper forms by peer workers at the site. Over a 7-week period, 32 visits were made to 9 saunas between 4 and 8 pm covering different days of the week. The number of visits (ranging from 1 to 8) for each sauna was determined after assessment by peer workers in conjunction with sauna operators on the number of clients, with the aim of recruiting all MSM above the age of 18 who agreed to participate in the study. Written informed consent was obtained from all participating MSM prior to the survey. An incentive, in the form of a HKD50 (USD1 = HKD7.8) cafeteria coupon, was offered on completion of each questionnaire.

Structurally, the questionnaire is divided into 3 main parts, with the first part covering demographics (age, ethnicity, employment, education level, residency in Hong Kong) and self-identified body appearance in accordance with the grouping of such characteristics proclaimed by MSM during field study – lean/lean toned; slim/slim/fit; sporty; muscular/macho; business suit; chubby; bear; feminine. Respondents were also divided into single type and mixed type appearance (table 1). In the second part, information about sex partners was profiled followed by a review on partner-sourcing channels. On partner characteristics, MSM were inquired about the number and types of sex partners, separated into steady partners or “lovers” (a term preferred by local respondents, denoting sex partner(s) that one considered to be emotionally attached); regular partners (those with sexual relationship without emotional attachment over extended period), casual partners (one-night-stand partners), and commercial partners (those exchanging sex for money or kind, also termed “money-boy”). Partner sourcing was defined as the networking of sex partners through specific channels; and respondents were asked if they had sourced partners at the following types of venues in the preceding 3 months – public toilet, bars, saunas, parties and/or sex parties and different frequency. They were also inquired if they had sourced partners through internet (chatroom, instant messaging and presence (IMP), bulletin board system (BBS) and smart-phone, the latter referring to special applications (Apps) for networking. In the last part, MSM were asked about the number of sex partners sourced through the various channels in the preceding 3 months. The frequency of condom use for anal and oral sex with the four types of partners was assessed for the preceding 3 month period. Condom use was ranked at 4 levels – never, sometimes (less than half of the occasions), frequently (more than half the occasions), and always (100%).

**Table 1:** General characteristics of all respondents (n = 299).

Characteristics		n	(%)
1. Employment status	Self-employed	24	(8.0)
	Full-time	204	(68.2)
	Part-time	18	(6.0)
	Student	47	(15.7)
	Unemployed	5	(1.7)
	Others (e.g. retired)	1	(0.3)
2. Education level	Primary	2	(0.7)
	Secondary	109	(36.5)
	Tertiary	188	(62.9)
3. Self-identified body appearance	(a) Lean or Lean toned	102	(34.1)
	(b) Slim or Slim fit	92	(30.8)
	(c) Sporty	78	(26.1)
	(d) Muscular/Macho	43	(14.4)
	(e) Business suit	40	(13.4)
	(f) Chubby	28	(9.4)
	(g) Bear	7	(2.3)
	(h) Feminine	1	(0.3)
4. Type of sex partners*	Steady partner	140	(46.8)
	Regular partner	79	(26.4)
	Casual partner	287	(96.0)
	Commercial partner	4	(1.3)
5. Partner-sourcing at (a) Physical venues	Public toilet	5	(1.6)
	Bar	112	(37.5)
	Sauna	292	(97.7)
	Group sex party	15	(5.0)
	Dance party	57	(19.1)
6. Partner-sourcing at (b) Virtual channels	Any form of internet	155	(51.8)
	Smart phone	90	(30.1)

*Type of sex partners in recent 3 months: steady partner – termed “lover” by the local community, which refers to sex partners that respondents are emotionally attached to and have been in a stable relationship; regular partners – partners with sexual relationship without emotional attachment over extended period; casual partners – one night-stand sex partners; commercial partners – those exchanging sex for money or kind, also termed “money-boy”.

In the analysis, the means of sourcing sex partners of MSM was distinguished into physical venues and virtual channels, the latter further subdivided into internet with or without the use of smart-phone, again in the previous 3 months. Comparison was made between users of physical venues alone and concomitant users of physical and virtual channels, and then between smart-phone users and non-smart phone users on their association, if any, with sex partner profile as well as the practice of unprotected sex in the 3 month period before interview. MSM were classified as “high risk” if they sometimes (less than half of the occasions) or never used condom for anal intercourse with any of their partners (steady, regular or casual partners), whereas those frequently (more than half of the occasions) or always using condom were considered “low risk”. Comparison was made by bivariate logistic regression. Statistical significance was two-sided and a threshold of p<0.05 was considered to be significant for all analyses. PASW Statistics 18 was used in performing statistical analyses.

## Results

### Characteristics of respondents and their partners

Between December 2010 and January 2011, a total of 572 MSM were approached, of which 326 (57%) had agreed to be interviewed. Data from 27 respondents were discarded since 8 had not have sex in the preceding three months and 19 had missing data on demographics, partner sourcing means and questions on risk behaviours. Data from a total of 299 respondents were finally evaluated. Table 1 outlines the demographics, self-identified body appearance, partner-sourcing means and partner profile. Participants in the study were all Chinese aged 18–56 years, with a median age of 30. About two-thirds had completed tertiary education (188/299, 62.9%) and most were currently employed (246/299, 82.3%). Eight options were given for participants to define their body appearance: a highest proportion self-identified as “lean or lean toned” (34.1%), followed by “slim or slim fit” (30.8%) and “sporty” (26.1%) whereas 10 (3.3%) considered themselves not belonging to any specific type. About 40% identified themselves with more than one type of body appearance and were referred as mixed type, e.g. lean and sporty.

Three main types of partners were reported by the respondents: steady (46.8%), regular partners (26.4%) and casual partners (96.0%). Only four MSM admitted having commercial partners or “money boy” (i.e. sex in exchange for money or kind), the number of which was too small for subsequent analyses. In the preceding 3 months, partner sourcing took place at either physical venues or settings, and/or virtual channels, the latter including internet and the use of smart-phone applications. All virtual channel users had frequented saunas, as this was the location where the study was conducted. Overall, all except one had visited one or more of the various physical venues for partner-sourcing, of which a majority (98.0%) sourced partners at saunas. Some 60.9% used internet on top of physical venues for partner-sourcing (12.7% for chatroom; 37.1% for IMP; 28.4% for BBS). Ninety (30.1%), all of which internet users, had used smart-phone for sourcing sex partners. Apart from saunas, bar (37.5%) was the most popular physical venue for partner-sourcing. For those with steady and/or regular partners, the number of respective partners was 1 or 2 in almost all cases. The number of casual partners was more varied: 1–2 (n = 76); 3–5 (n = 122); 6 or more (n = 90). Among the 299 subjects, a total of 1554 sex partners had been successfully networked in the preceding 3 months, of which 87.1% were acquainted in saunas.

### Comparing between users of different partner sourcing means

Of the 299 MSM interviewed, 2 broad groups could be distinguished by their means of partner-sourcing: sauna users (n = 78), and concomitant users of saunas and virtual channels (n = 179).(Table 2(a)) Respondents who had used virtual channels (any form of internet or smart-phone) were generally younger (median age of 28 vs. 35, *p*<0.001) than sauna users who did not use internet. No specific body appearance was dominant by channel of sourcing though a higher proportion of those using virtual channels self-identified themselves with more than one type of body appearance, but the difference did not reach statistical significance (mixed type, 25.1% vs. 24.4%, OR = 1.65, *p* = 0.105). Overall, public toilets and sex parties were uncommonly used for partner sourcing, as reported by less than 1% of respondents. There was no difference in the total number of sex partners acquainted in the preceding 3 months between MSM using physical channel alone and concomitant users of physical/virtual channels, and this remains the same after separating into different types of sex partners, i.e. steady, regular and casual partners (results not shown).

**Table 2:** Comparison of characteristics between (a) sauna users versus virtual+sauna users, and (b) smart-phone and non smart-phone internet users.

	(a) virtual and sauna users (n = 179) versus sauna users (n = 78)							(b) smart phone users (n = 90) versus non smart phone but internet users (n = 93)						
	Virtual channels users		Physical venues users		OR	95% CI	*p*-value	Smart- phone users		Non-smart phone but internet users		OR	95% CI	*p*-value
(A) General characteristics														
Age, in median (IQR)	28	(8.0)	35	(12..0)	-	-	<0.001	27	(9.0)	30	(10.5)	-	-	0.094
Education level, n (%)														
Secondary or below	63	(35.2)	35	(44.9)	0.67	(0.39, 1.15)	0.143	30	(33.3)	33	(35.5)	0.91	(0.49, 1.67)	0.760
Tertiary	116	(64.8)	59	(75.6)	-	-	-	60	(66.7)	60	(64.5)	-	-	-
Self-identified appearance§ n (%)														
Mixed type	62	(25.1)	19	(24.4)	1.65	(0.90, 3.00)	0.105	35	(38.9)	29	(31.2)	1.40	(0.76, 2.59)	0.275
Single type	117	(65.4)	59	(75.6)				55	(61.1)	64	(68.8)	-	-	-
(C) Frequently used physical venues for partner-sourcing¶ n(%)														
*Bar*	-	-	-	-	-	-	-	24	(26.7)	22	(23.7)	1.17	(0.60, 2.29)	0.639
*Sauna*	-	-	-	-	-	-	-	53	(58.9)	58	(62.4)	0.86	(0.48, 1.57)	0.630
*dance party*	-	-	-	-	-	-	-	16	(17.8)	8	(8.6)	2.30	(0.93, 5.67)	0.071
(C) Total numbers of sex partners, in median (IQR)	4	(3.0)	4	(4.0)	-	-	0.654	4	(4.0)	5	(3.0)	-	-	0.687

§“Single type” refers to only one self-identified appearance (refer to item 3 in Table 1) whereas “mixed type” refer to a combination of different types of self-identified appearance.

¶Frequent users are defined as using the channels from once weekly to once daily.

To further explore potential differences among users of virtual channels, the latter were divided into smart-phone users (n = 90) and non smart-phone internet users (n = 93). These two groups had similar age, education level and self-identified body appearance as distinguished into mixed or single body type. There was however, a higher proportion of “lean/lean toned” (45.6% vs. 31.2%, p<0.05) among smart-phone users. There were otherwise no statistically significant differences in the number of different types of sex partners and the total numbers of sex partners between the two groups(Table 2(b)). 

### Partner sourcing means and sexual risk

We evaluated the consistency of condom use in MSM for anal and oral sex with 3 respective types of sex partners (i.e. steady, regular and casual) in the 3 month period prior to the interview. Overall, a high proportion (97.4%) had always or frequently used condom for anal sex with casual partners, but not with steady partners (33.9%) or regular partners (66.2%). The use of condom for oral sex was universally low. There was no difference in the consistency of condom use between sauna users and sauna/virtual channel users. (Table 3) We then compared the sexual risk of HIV/STI infections in MSM in connection with their means of partner sourcing (Table 4). There were 102 “high risk” (n = 102) MSM who sometimes (less than half of the occasions) or never used condom for anal intercourse with any of their partners (steady, regular or casual partners), while 182 could be defined as having “low risk” as they had always or frequently (more than half of the times) used condom. In the “high risk” category, more MSM had sourced sex partners in bar (25.5% vs. 14.3%, OR = 2.05, p<0.05). Some body appearance types were commoner in “high risk” compared to “low risk” category: slim/slim fit, muscular/macho and business suit. Users of virtual channels tended to have higher sexual risk compared with users of physical venues (71.6% vs. 58.2%, OR = 1.81, p<0.05), but there was no difference in the proportion of smart-phone users between the two categories.

**Table 3:** Condom use for anal and oral sex with different types of sex partners in the study population.

Sexual practice	Partner type	Consistent condom use§ no.(%)		*p*-value (Chi Square test)
		Sauna and virtual channel users	Sauna users	
Anal	Steady (n = 103)	23 (29.1%)	12 (50%)	0.058
	Regular (n = 59)	30 (63.8%)	8 (66.7%)	1.000#
	Casual (n = 237)	164 (97.0%)	67 (98.5%)	0.676#
Oral	Steady (n = 111)	0 (0%)	2 (7.1%)	0.062#
	Regular (n = 60)	1 (2.1%)	1 (7.7%)	0.389#
	Casual (n = 239)	4 (2.4%)	8 (11.3%)	0.007# *

§ Respondents who always (every time) or frequently (more than 50% of the time) used condom when having sex with the respective partners in the preceding 3 months.

# Fisher's Exact Test was used as at least 1 cell had an expected count of less than 5.

*p<0.05.

**Table 4:** Characteristics of respondents with high-risk sexual behaviour (n = 102) versus low risk (n = 182).

		High risk#		Low risk#		OR	95% CI	*p*-value
(a) General characteristics								
Age, in median (IQR)		31	(9)	30	(12)	-	-	0.208
Education level, n (%)	Secondary or below	33	(32.4)	70	(38.5)	0.77	(0.46, 1.28)	0.305
	Tertiary	69	(67.6)	112	(61.5)	-	-	-
Self-identified appearance# #, n(%)								
Overall	Lean or Lean toned	41	(40.2)	60	(33.0)	1.37	(0.83, 2.26)	0.223
	Slim or Slim fit	20	(19.6)	62	(34.1)	0.47	(0.27, 0.84)	0.011*
	Sporty	30	(29.4)	46	(25.3)	1.23	(0.71, 2.12)	0.450
	Muscular or Macho	20	(19.6)	20	(11.0)	1.98	(1.01, 3.88)	0.048*
	Business suit	21	(20.6)	19	(10.4)	2.22	(1.13, 4.37)	0.020*
Complexity	Mixed type	37	(36.3)	52	(28.6)	1.42	(0.85, 2.38)	0.180
	Single type	65	(63.7)	130	(71.4)	-	-	-
(b) Partner-sourcing means								
Frequently used venues¶ for partner-sourcing								
	Bar	26	(25.5)	26	(14.3)	2.05	(1.12, 3.77)	0.021*
	Sauna	59	(57.8)	101	(55.5)	1.10	(0.67, 1.80)	0.702
	dance party	12	(11.8)	16	(8.8)	1.38	(0.63, 3.05)	0.422
User of virtual and physical means								
	Virtual means	73	(71.6)	106	(58.2)	1.81	(1.07, 3.04)	0.026*
	Physical means§	29	(28.4)	76	(41.8)	-	-	-
User of virtual means								
	Smart phone	30	(41.1)	57	(53.8)	0.60	(0.33, 1.10)	0.096
	Non smart phone but internet	43	(58.9)	49	(46.2)	-	-	-

# High-risk: Respondents sometimes (less than 50% of the time) or never used condom when having anal sex with steady, regular or casual partners in the preceding 3 months; Low risk: Respondents always (every time) or frequently (more than 50% of the time) used condom when having anal sex with steady, regular and casual partners in the preceding 3 months.

# # “Single type” refers to only one self-identified appearance (refer to item 3 in Table 1) whereas “mixed type” refers to a combination of different types of self-identified appearance.

¶ Frequent users are defined as those using the channel from once weekly to once daily.

§ Physical venues: public toilet, bar, sauna, sex party and dance party.

*p<0.05.

## Discussion

In this study we have characterized sauna-visiting MSM by their main channel of sex partner sourcing, namely physical venue (largely saunas) alone and the use of both physical and virtual channels, the latter centering on internet and extending to smart-phone use. To the best of our knowledge, this is the first study exploring partner-sourcing and STI-risk behaviors among smart-phone users in the MSM communities. Our results suggest that virtual channels users were also frequent users of a range of other physical venues (e.g. bar, sauna and dance party). The convenience of the internet has enabled MSM to concurrently network actively through physical venues, thereby predisposing to engagement in high risk activities. Consistent with previous studies, we confirmed that the use of virtual channels was associated with a higher prevalence of unprotected anal intercourse [13]–[16]. Elsewhere, studies have suggested that internet increases HIV/STI risk by enhancing efficiency of initiating sexual liaisons [22]. One recent study reported that high-risk sexual behaviours were practised between MSM with both online and offline partners and MSM with only online or offline partners [23]. The results in our study are consistent with this observation in that MSM with high-risk sexual behaviour were the frequent users of bar for partner-sourcing as well as being virtual channels users. By the same token, it can be speculated that smart-phone users may be at higher HIV/STI risk as the mobile device would enable users to get connected with other MSM efficiently at anytime and in any place, especially so when GPS application is activated. The continued rise in the number of smart-phone users and the frequent use of sex networking applications (“Apps”) may change the scenario in the coming years.

Finding in our study did not support any additional risk associated with smart-phone users compared with non smart-phone internet users, both in terms of engaging in high-risk sex behaviours and the number of sex partners. Whether the popularity of smart-phone use would ultimately lead to changes in the configuration of MSM network is not known. Currently smart-phones have certainly enhanced the efficiency of social networking among the MSM, but it is not associated with any difference in HIV/STI-risk behaviours. As the proportion of smart-phone users remained low at 30% of all internet users in our sample, it could be argued that the impacts have not yet been felt. At the current point of time, the findings in this study do raise important questions on the future strategies of HIV/STI prevention in the MSM community. In the past, it sufficed for safer sex promotion activities to be organized at physical venues like saunas and settings like parties which were popular among MSM. The widespread coverage of internet has shifted attention of public health programmes to target virtual channels, the same channels used by MSM for potentially higher risk sexual activities. Over the years, new efforts have been made involving the use of internet as vehicles to disseminate HIV prevention messages and interventions [24]. Unlike physical channel users who may have sex at the venues, virtual channel users are more inclined to have sex at home, an environment where community prevention efforts may be hard to penetrate. It is therefore crucial to track the changing dynamics of sex partnership so that tailored intervention can be developed targeting people with higher risk of HIV/STI transmission. Finally, smart-phones appeared to be favoured by selected groups of MSM in our study, as reflected by a higher proportion of some respondents with specific body types. The phenomenon of assortative mixing has been suggested between partners of similar age, and same race/ethnicity, while that for body types has not been explored [25], [26]. Extrapolation of the results in the MSM community is however cautioned, and further research is needed to explore the association between sexual networking and body appearance.

The primary limitation of our study was the venue-based sampling strategy. The survey was conducted in saunas frequently visited by MSM in Hong Kong and hence, generalization to all MSM or MSM at other sites cannot be assumed. Likewise, direct comparison between virtual channel and physical channel for sex partner sourcing was not possible as all respondents have been recruited from a physical channel for partner sourcing (i.e. saunas). At best we could compare between sauna using MSM and those engaged in concomitant sauna and virtual channel use. On the other hand, the study was conducted when smart-phone has just begun (late 2010) to emerge as a popular means for partner-sourcing. The use of smart-phone and its integration with social media is increasing the complexity of the networking patterns of MSM, who are also more likely to frequent social venues like bars for sourcing sex partners. The demographic characteristics and sexual behaviours of smart-phone users may evolve over time and therefore further studies are warranted as smart-phone may eventually become one dominant means of sex networking in future.

The surge of smart-phone users reminds us of the need to evaluate the impacts of virtual channels for sex-networking in MSM, and to examine if this modern device is associated with a higher potential risk of HIV/STI transmission in MSM.
